# Dendritic Cell Profile Induced by *Schistosoma mansoni* Antigen in Cutaneous Leishmaniasis Patients

**DOI:** 10.1155/2014/743069

**Published:** 2014-09-17

**Authors:** Diego Mota Lopes, Jamille Souza Fernandes, Thiago Marconi de Souza Cardoso, Aline Michele Barbosa Bafica, Sérgio Costa Oliveira, Edgar M. Carvalho, Maria Ilma Araujo, Luciana Santos Cardoso

**Affiliations:** ^1^Serviço de Imunologia, Complexo Hospitalar Universitário Professor Edgard Santos, Universidade Federal da Bahia, Rua João das Botas s/n, Canela, 40110-160 Salvador, BA, Brazil; ^2^Instituto Nacional de Ciência e Tecnologia de Doenças Tropicais (INCT-DT/CNPq), Salvador, BA, Brazil; ^3^Departamento de Bioquímica e Imunologia, Universidade Federal de Minas Gerais, Belo Horizonte, 31270-901, MG, Brazil; ^4^Escola Bahiana de Medicina e Saúde Pública, 40050-420 Salvador, BA, Brazil; ^5^Departamento de Análises Clínicas e Toxicológicas, Faculdade de Farmácia, UFBA, 40170-115 Salvador, BA, Brazil

## Abstract

The inflammatory response in cutaneous leishmaniasis (CL), although responsible for controlling the infection, is associated with the pathogenesis of disease. Conversely, the immune response induced by *S. mansoni* antigens is able to prevent immune-mediated diseases. The aim of this study was to evaluate the potential of the *S. mansoni* Sm29 antigen to change the profile of monocyte-derived dendritic cells (MoDCs) from subjects with cutaneous leishmaniasis (CL) *in vitro*. Monocytes derived from the peripheral blood mononuclear cells of twelve patients were cultured with GM-CSF and IL-4 for differentiation into dendritic cells and then stimulated with soluble *Leishmania* antigen (SLA) in the presence or absence of Sm29 antigen. The expression of surface molecules associated with maturation and activation (HLA-DR, CD40, CD83, CD80, and CD86), inflammation (IL-12, TNF), and downregulation (IL-10, IL-10R) was evaluated using flow cytometry. We observed that the frequencies of HLA-DR, CD83, CD80, and CD86 as well as of IL-10 and IL-10R on MoDCs were higher in cultures stimulated with Sm29, compared to the unstimulated cell cultures. Our results indicate that the Sm29 antigen is able to activate regulatory MoDCs in patients with cutaneous leishmaniasis. It might be useful to control the inflammatory process associated with this disease.

## 1. Introduction

Leishmaniasis is endemic in 88 countries with approximately 12 million people infected and 350 million at risk worldwide, with an incidence of 1.5 million cases per year [1, 2]. A variety of disease manifestations are associated with* Leishmania *spp. infection, primarily determined by the infecting species. Cutaneous leishmaniasis (CL) is the most common clinical manifestation of tegumentary leishmaniasis, characterized by one to several skin lesions in exposed areas, with small number of parasites [3, 4]. The immune response is characterized by a Th1-inflammatory profile with macrophage activation and parasite killing. During the innate immune response monocyte-derived dendritic cells (MoDCs) which interact with the pathogen may differentiate at the inflammation site and act as local tissue resident APCs or as a source of inflammatory cytokines [5, 6]. The initial events determine the cytokine environment and consequent adaptive immune response to certain pathogens, such as* Leishmania*. For instance, this parasite leads to an exacerbated Th1-inflammatory immune response associated with tissue injury in LC. On the other hand, the immune response induced by the parasite* Schistosoma mansoni* is able to downregulate the inflammatory response in immune-mediated diseases [7–11]. Studies have shown that chronic helminths infections, especially* Schistosoma mansoni*, possess the ability to modulate the inflammatory response associated with both Th1 [7, 9] and Th2 [8, 11–13] immune-mediated diseases. These findings have provided the rationale for the use of recombinant* S. mansoni* proteins in* in vitro* studies with cells from patients with leishmaniasis in an attempt to modulate the inflammatory response associated with pathogenesis. Previous studies performed by our group have shown that the addition of the* S. mansoni* antigens Sm29, PIII, and TSP-2 in cultured PBMC from cutaneous leishmaniasis patients stimulated with soluble* Leishmania* antigen (SLA) caused a reduction in the levels of IFN-*γ* and TNF in a significant number of individuals, with an increase in the levels of IL-10 [9]. In other studies from our group [7, 9, 11] Sm29 has been the better inducer of IL-10 among the* S. mansoni* antigens tested, a cytokine with the property to prevent inflammatory process associated with immune-mediated diseases. The aim of this study was to evaluate the potential of the* S. mansoni* antigen Sm29 to induce a regulatory profile in monocyte-derived dendritic cells (MoDCs) from individuals with CL in an attempt to prevent or minimize the inflammatory response associated with the disease.

## 2. Material and Methods

### 2.1. Study Design

We included twelve patients with CL who reside in an endemic area, named “Corte de Pedra,” located in the southeast region of the state of Bahia, Brazil. Six of them were male and six female with a mean age of 33 ± 6 years. The diagnostic criteria comprised a clinical presentation characteristic of CL, parasite isolation or positive delayed-type hypersensitivity (DTH) in response to* Leishmania* soluble antigen (SLA), and histological features of CL.

The Ethics Committee of the University of the State of Bahia (UNEB) approved the present study (License Number 0603110287514). Three stool samples from each individual were examined using the Hoffman sedimentation method to exclude individuals infected with* S. mansoni*.

### 2.2. Antigen Stimulation

The* Schistosoma mansoni* tegument antigen Sm29 used in this study was provided by Dr. Sergio C. Oliveira from the Institute of Biological Science, Department of Biochemistry and Immunology, UFMG, Brazil. The recombinant proteins were cloned in* E. coli* and were tested for the presence of lipopolysaccharide (LPS) using a commercially available LAL Chromogenic Kit (CAMBREX). The level was below the detection limit (data not shown). The SLA was prepared from a* L. braziliensis* strain as previously described [14].

### 2.3. *In Vitro* Generation of Monocyte Dendritic Cells (MoDCs)

MoDCs were obtained from PBMCs from cutaneous leishmaniasis patients as reported in previous studies [15]. Briefly, PBMCs were obtained from the Ficoll-Hypaque gradient method and cultured in 6-well plates with complete medium RPMI 1640 medium containing 10% inactivated fetal bovine serum, 100 mg/mL gentamicin, 2 mM L-glutamine, and 30 mM HEPES (Gibco-BRL Life Technologies, Gaithersburg, MD) at a concentration of 5 × 10^6^ cells per well. They were incubated for 2 hours with 5% CO_2_ at 37°C to allow for monocyte adherence to the plate. After this period, the supernatant was removed, and cells were washed three times with PBS to remove nonadhered cells. The adhered monocytes were harvested with a solution containing PBS1x, EDTA (10 nM), and glucose (3 nm) and then adjusted to 3 × 10^5^ cells/mL and cultured with complete medium in the presence of IL-4 (800 IU/mL) and GM-CSF (50 ng/mL) for 6 days with the replacement of medium and cytokines at day 3 to allow for differentiation into dendritic cells. Subsequently, 3 × 10^5^ MoDCs were cultured with Sm29 (10 *μ*g/mL) and SLA (5 *μ*g/mL) for 20 hours. At this point, the MoDCs were assessed by flow cytometry. Next, we determined the percentage of CD11c^+^ cells by flow cytometry, and for all experiments, the frequency of these cells was ≥80%.

### 2.4. Flow Cytometry

For flow cytometry, MoDCs were harvested and stained with fluorochrome-conjugated antibodies for surface and intracellular markers. DCs treated with different antigens or controls (without stimulation) were collected by centrifugation at 1100 rpm for 10 min and resuspended in RPMI 1640 containing 10% fetal bovine serum (FBS, heat inactivated) (GIBCO, INVITROGEN). Cells were stained with fluorescently conjugated mouse anti-human monoclonal antibodies against CD11c-APC (clone 3.9), CD1a-FITC (clone HI149), IL-10R*α*-PE (polyclonal), CD40-PerCP-e Fluor 710 (clone 5C3), CD80-PerCP-e Fluor 710 (clone 2D10.4), CD86-PE (clone IT2.2), CD83-PE-Cy7 (clone HB15e), and HLA-DR-PerCP-Cy5.5 (clone LN3) (all from eBioscience, California) and then analyzed for 100000 events per sample using a flow cytometer (FACSCanto, Becton Dickinson). Limits for the quadrant markers were set based on negative populations and controls isotype (data not shown).

Intracellular staining was performed with PE-labeled monoclonal antibody against human IL-10 (clone JES3-19F1, BD Pharmingen), IL-12 (clone C8.6, eBioscience), and TNF (clone Mab11, eBioscience) in saponin buffer (PBS, supplemented with 0.5% BSA and 0.5% saponin). During the last 4 hours of culture, Brefeldin A (10 *μ*g/mL; Sigma, St. Louis, MO) was added to the cultures. Afterwards, the cells were washed in PBS and fixed in 2% formaldehyde for 20 minutes at room temperature.

**Figure 1:**
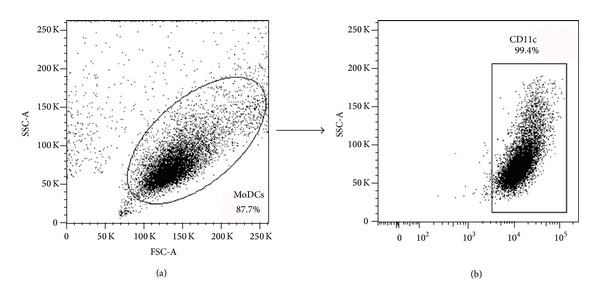
Gate strategy for the identification of monocyte-derived dendritic cells (MoDCs) (a). Frequency of cells expressing CD11c (b). Representative graph of one experiment.

The frequency of positive cells was analyzed using the program FlowJo (Tree Star, USA) in two regions. The monocyte-derived dendritic cell region was defined by nonspecific fluorescence with forward scatter (FSC) and side scatter (SSC) as parameters of cell size and granularity, respectively. The cells were gated based on their granularity and expression of CD11c (Figure 1).

### 2.5. Statistical Analysis

Data were analyzed using GraphPad Prism 5.0 (GraphPad Software, San Diego, CA, USA). The differences among MoDCs stimulated with SLA in the presence or absence of Sm29 antigen were assessed using Friedman exact test. The frequencies of positive adherents cells were expressed as percentages and mean fluorescence intensity (MFI), respectively. Statistical significance was established at the 95% confidence interval.

## 3. Results

### 3.1. Frequency and Maturation Status of Monocyte-Derived Dendritic Cells (MoDCs) Stimulated with the* S. mansoni* Antigen Sm29

**Figure 2:**
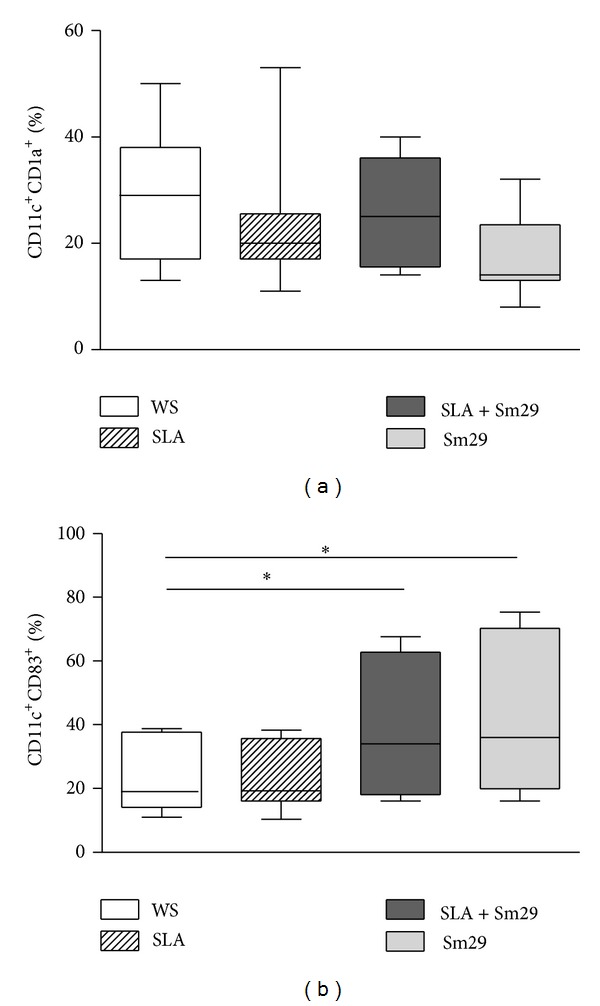
Maturation status of MoDCs from CL patients stimulated with Sm29 antigen. Frequency of MoDCs expressing CD1a (a) and CD83 (b) from individuals with CL (*n* = 12). WS = without stimulation, SLA = soluble* Leishmania *antigen, and Sm29 =* S. mansoni* Sm29 antigen. The results were expressed as median, min-max values, and percentiles. *P < 0.05, Friedman test.

The frequency and maturation status of MoDCs stimulated with SLA in the presence or absence of Sm29 were evaluated* in vitro* by the expressions of CD1a and CD83 molecules on CD11c^+^ cells (Figure 2). The addition of Sm29 to SLA stimulated cultures did not alter the frequency of the CD1a molecule on MoDCs (Figure 2(a)). The frequency of CD11c^+^CD1a^+^ cells was similar among cultures stimulated with SLA (98% (93–99%)), SLA + Sm29 (98% (90–99%)), and Sm29 alone (97% (92–99%)). Regarding the maturation status evaluated through the expression of CD83 molecule, it was observed that the addition of Sm29 to the cultures of MoDCs leads to an increase in the frequency of CD83, being 27.5% (16–68%) in SLA + Sm29 cultures and 42 (16–75%) in cultures with Sm29 alone compared to cultures without stimulation (19% (11–39%), *P* < 0.005, Figure 2(b)).

### 3.2. Activation Status of MoDCs after Addition of Antigen Sm29

**Figure 3:**
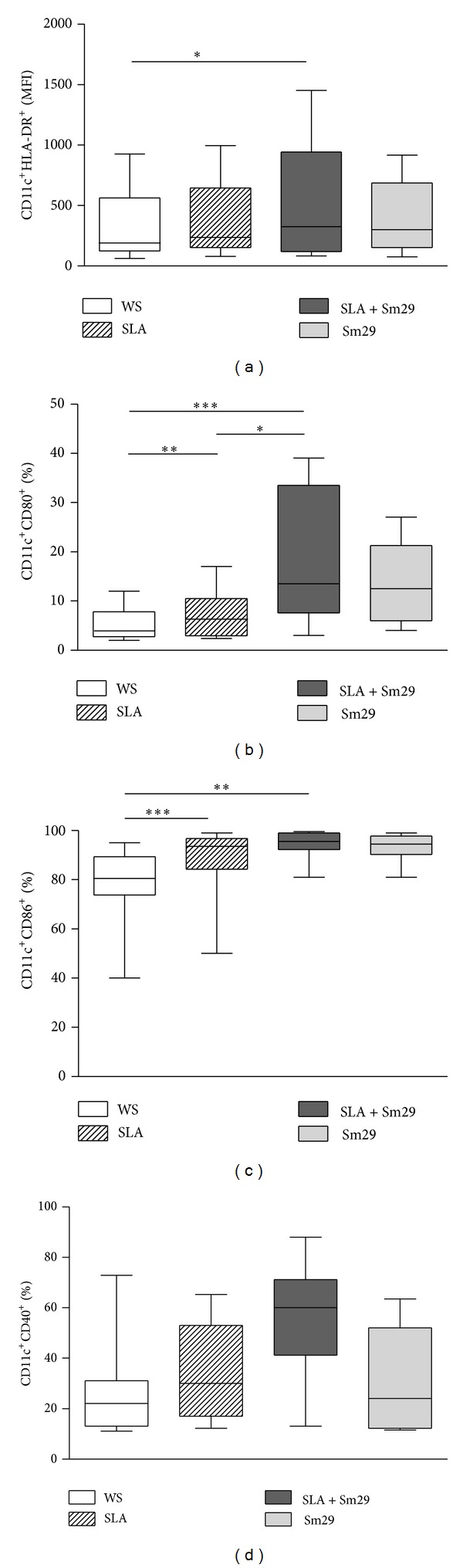
Activation status of MoDCs. Mean florescence intensity of the expression of HLADR (a) and frequency of MoDCs expressing CD80 (b), CD86 (c), and CD40 (d) in cell cultures from patients with cutaneous leishmaniasis (*n* = 12). WS = without stimulation. SLA = soluble* Leishmania *antigen. Sm29 =* S. mansoni* antigen Sm29. The results are expressed as median, min-max values, and percentiles. *P < 0.05, **P < 0.005, and ***P < 0.001, Friedman test.

The mean fluorescence intensity (MFI) of activation marker HLA-DR on MoDCs after* in vitro* addition of Sm29 in cultures stimulated with SLA was also evaluated (Figure 3(a)). The addition of Sm29 antigen in cultures stimulated with SLA leads to an increase in the expression of HLA-DR by MoDCs (325 (83–1452) MFI) when compared to the unstimulated cultures (185 (62–927) MFI, *P* < 0.05; Figure 3(a)). The expression of costimulatory molecules CD80, CD86 was also affected by the presence of antigens (Figures 3(b) and 3(c)). The addition of Sm29 to the cultures stimulated with SLA led to an increase in the frequency of cells expressing CD80 (13.5% (3–39%)) compared to unstimulated cultures (3.9% (2–12%), *P* < 0.0001) or those stimulated with SLA alone (6.3% (2.4–17%), *P* < 0.05; Figure 3(b)). Regarding the frequency of MoDCs expressing CD86, the addition of Sm29 to the cultures stimulated with SLA showed an increase in the frequency of these cells (95.5% (81–99.5%)) compared to unstimulated cultures (80.5% (40–95%), *P* < 0.005; Figure 3(c)). The frequency of MoDCs expressing CD86 was also higher in cultures stimulated with SLA (93.5% (50–99%)) compared to unstimulated cultures (Figure 3(c)). The frequency of MoDCs expressing CD40 was similar among the groups, being 25.25% (11.10–72.90%) in cultures without stimulation, 31.35% (11.5–63.5%) for cultures stimulated with Sm29, 36.55% (12.2–65.3%) for cultures stimulated with SLA, and 56% (13–88%) in cultures stimulated in the presence of SLA plus Sm29 (Figure 3(d)).

In cultures stimulated with LPS there was an increase in the frequency of cells expressing CD40 (60.1% (45.3–71.8%)), compared to cultures without stimulus (25.25% (11.10–72.90%), *P* < 0.05) or stimulated with SLA (36.55% (12.2–65.3%), *P* < 0.05). The mean fluorescence intensity HLA-DR was higher in cultures with LPS (352 (116–2097) MFI) compared to the unstimulated cultures (82 (55–97) MFI, *P* < 0.05), data not shown.

### 3.3. Inflammatory and Regulatory Status of MoDCs Induced by Sm29

**Figure 4:**
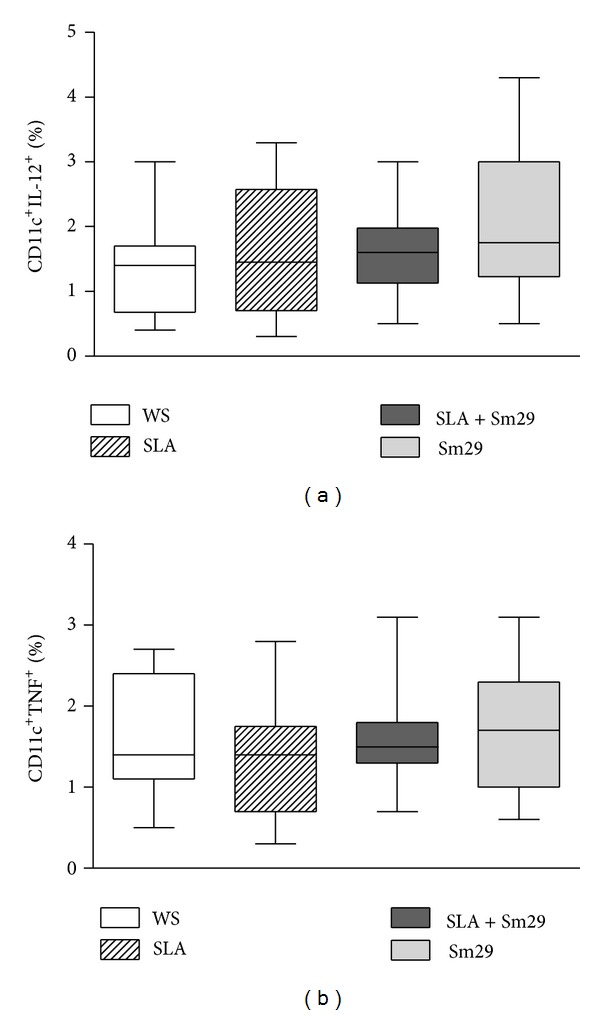
Inflammatory cytokines expressed by MoDCs from patients with cutaneous leishmaniasis (*n* = 12). Frequency of MoDCs expressing IL-12 (a) and TNF (b). WS = without stimulation. SLA = soluble* Leishmania *antigen. Results were expressed as median (min-max values and percentiles).

The expression of inflammatory cytokines IL-12 and TNF by MoDCs from patients with cutaneous leishmaniasis induced by the presence of Sm29 is shown in Figures 4(a) and 4(b). The frequency of MoDCs expressing IL-12 did not differ among all stimulated cultures, being 1.4% (0.4–3.05%) in cultures without stimulation, 1.45% (0.3–3.3%) in cultures stimulated with SLA alone, 1.6% (0.5–3.0%) in cultures stimulated with SLA + Sm29, or 1.75% (0.5–4.3%) in cultures stimulated with Sm29 (Figure 4(a)). The frequency of the MoDCs expressing TNF was also similar among all groups, being 1.45% (0.5–2.7%), 1.75% (0.6–3.1%), 1.40% (0.3–2.8%), 1.5% (0.7–3.1%), or 0.8% (0.4–2.7%) in the cultures without stimulation or stimulated with SLA, SLA + Sm29, or Sm29 alone, respectively (Figure 4(b)).

**Figure 5:**
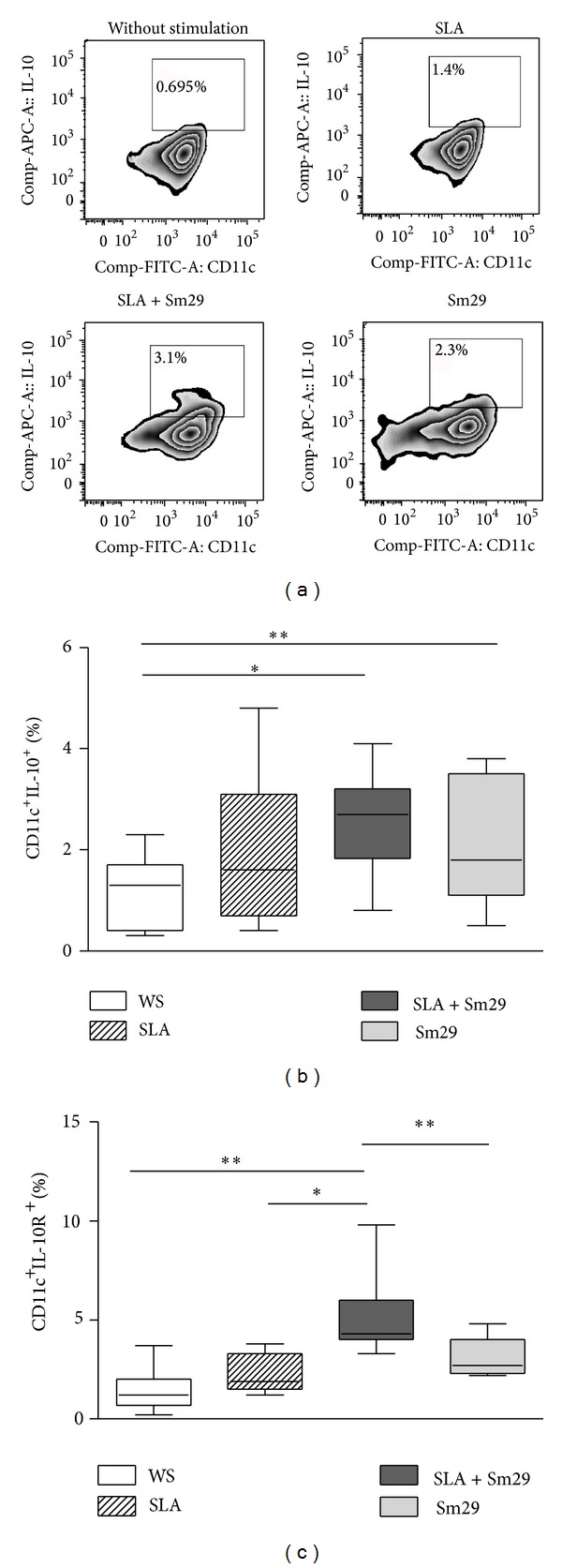
Regulatory markers induced by Sm29 antigen on MoDCs. Frequency of MoDCs expressing IL-10 ((a)-(b)) and IL-10R (c) in individuals with cutaneous leishmaniasis (*n* = 12). WS = without stimulation. SLA = soluble* Leishmania *antigen. Results were expressed as median (min-max values and percentiles). *P < 0.05 and **P < 0.005, Friedman test. (A) Representative plot of one experiment.

It has been demonstrated that the balance between inflammatory and regulatory networks is important to control the parasites and suppress the clinical manifestation of disease as observed in subclinical forms [16]. We then assessed the ability of Sm29 antigen to induce a regulatory profile by the MoDCs from patients with CL. The frequencies of MoDCs expressing the regulatory cytokine IL-10 in cultures stimulated with SLA + Sm29 (2.3% (0.8 to 4.1%)) or with Sm29 alone (1.8% (from 0.5 to 3.8%)) were higher when compared to the unstimulated cultures 1.3% (0.3–2.3%, *P* < 0.05; Figures 5(a) and 5(b)). Regarding the expression of IL-10 receptor (IL-10R) by MoDCs, we observed that in cultures stimulated with SLA + Sm29 there was a higher frequency of cells expressing this molecule (4.3% (3.3–9.8%)) compared to unstimulated cultures (1.4% (0.2–3.7%), *P* < 0.005) and cultures stimulated with SLA alone (2.5% (1.2–3.8%)) and with Sm29 alone (2.7% (2.2–4.8%); Figure 5(c)).

In cultures stimulated with LPS there was a reduction in the frequency of IL-10 (0.5% (0.3–1.2%), *P* < 0.005) and IL-10R (1.3% (1.0–1.8%), *P* < 0.05) when compared to cultures stimulated with SLA + Sm29 (*P* < 0.05). There was no significant difference in the frequency of MoDCs stimulated with LPS regarding the expression of IL-12 or TNF compared to unstimulated cells (data not shown).

## 4. Discussion

Cutaneous and mucosal leishmaniasis diseases result from the exacerbation of the Th1-inflammatory immune response. Additionally, the Th1 response with the production of IFN-*γ* and TNF represents the most important mechanism of* Leishmania* elimination by the activation of macrophages. However, once exacerbated, this response is associated with tissue damage, resulting in cutaneous and mucosal leishmaniasis [17]. The early events in* Leishmania* sp. infection involve dendritic cells and cytokine production, determining the host response and the course of the infection [18]. Thus, the cellular environment associated with the proinflammatory and anti-inflammatory balance is important to control parasite growth and prevent damage to the host [19–22]. This is observed in subclinical forms of* Leishmania* infection in endemic areas [16, 23].

There are evidences in the literature that infection with* Schistosoma *sp. or its products protects against the development of Th1 and Th2 mediated diseases as reviewed by Khan and Fallon (2013) and Elliott et al. (2007) [24, 25]. We have studied the relationship between inflammatory diseases and schistosomiasis in an attempt to characterize the ability of* S. mansoni *antigens to modulate the inflammatory process associated with immune-based diseases, such as asthma [11, 13], HTLV-1 [7], and leishmaniasis [9, 10]. We are currently interested in characteristics of parasite antigens with modulatory properties, able to downmodulate the inflammatory process in CL patients. The mechanisms underlying the regulatory property of* S. mansoni* antigens may include induction of cells and regulatory molecules, such as CD4^+^CD25^+^ T cells, CTLA-4, and IL-10 molecules [9, 11, 13, 26–29].

In a study performed by our group, Bafica and colleagues (2011) showed that the addition of Sm29 antigen to the cultures of PBMC from CL patients resulted in an increase of IL-10 production in a significant number of patients, coincidental with a reduction in the production of the inflammatory cytokines TNF and IFN-*γ*. Since in leishmaniasis dendritic cells seem to be important to orchestrate the initial immune response, the use of an* S. mansoni* antigen able to modulate the inflammatory response of these cells is an important strategy to control the exacerbated response observed in patients with cutaneous leishmaniasis.

The addition of Sm29 to the cultures did not alter the frequency of MoDCs expressing CD1a^+^. It is in agreement with a study performed by Donovan et al. (2007) that showed that infection by* L. major* or* L. donovani* was capable of inhibiting the expression of CD1a^+^ in dendritic cells, decreasing their ability to recognize pathogens and thus respond to their stimuli [30]. Moreover, human monocytes in the presence of* L. amazonensis* showed a decreased expression of CD1a* in vitro* leading to an incomplete differentiation into dendritic cells [30].

Studies have shown that infection by helminths or the use of parasite antigens in* in vitro* studies results in low rate of dendritic cell differentiation, low CD1a expression, and impaired maturation status due to decreased expression of CD83 in MoDCs, both in individuals infected with helminths and in healthy controls [31, 32].

When we assessed the influence of Sm29 antigen on the expression of CD83 on MoDCs, we observed an increase in the frequency of this marker in cells in the presence of this antigen. Different pathogens can influence the maturation and activation status of dendritic cells and affect the outcome of infection [33, 34]. Terrazas et al. (2010) showed that the addition of an antigen of* T. crassiceps* (TCEs) to the cultures stimulated with LPS led to an increase in the expression of CD83 by MoDCs. However, the presence of TCEs alone did not alter the maturation status of these cells. Favali et al. (2007) showed that infecting MoDCs with* L. amazonensis* or stimulation of these cells with SLA did not alter the maturation status of DCs [35]. The maturation process is essential to make DCs capable of presenting antigens to T cells, as well as increasing their ability to produce cytokines. Studies have shown that the exposure of DCs to parasite antigens, including those derived from helminths, results in a limited maturation of these cells [36, 37].

In studies with experimental models, an increase was demonstrated in the frequency of activation markers (HLADR, CD80, and CD86) on dendritic cells infected with* Leishmania *sp. [38, 39]. However, Carvalho et al. (2008) showed that bone marrow-derived DCs from uninfected mice (bystander) present in the environment of DCs infected with* L. braziliensis* showed a higher activation status compared to those cells infected with* L. braziliensis*. It suggested that bystander dendritic cells are better responders to the parasite antigens than the infected ones [39]. Moreover, it has been demonstrated that SLA stimulation of MoDCs of healthy subjects infected with* L. amazonensis* does not alter the expressions of HLA-DR, CD80, and CD86 when compared to uninfected or unstimulated DCs [35].

In this study the frequency of MoDCs expressing CD40 did not alter in the presence of Sm29. A study conducted by Dowling et al. (2011) demonstrated that stimulation of DCs with the antigen ABF from* A. lumbricoides* also did not affect the frequency of the CD40 expression on these cells [40].

It is well known that the cytokines IL-12 and TNF have an important role in mounting the Th1-inflammatory response and control* Leishmania* infection. On the other hand, it is also known that an increased inflammatory response with high production of these cytokines is associated with the development of clinical manifestations of CL [17].

Since it has been shown that there is an impairment of IL-10 production by cells of CL patients [17] and that* S. mansoni* antigens induce the production of this cytokine [8, 9], we decided to evaluate the expression of IL-10 and its receptor on DCs stimulated with Sm29 antigen. We observed that the Sm29 antigen led to an increase in the frequency of MoDCs expressing IL-10 in patients with CL. It has been shown that IL-10 inhibits the differentiation of dendritic cells and suppresses the production of inflammatory chemokines and cytokines [41, 42]. A balance in the immune response, where activated macrophages continue to kill* Leishmania*, without harm to the host is desirable. In this context, DCs stimulated with Sm29 antigen could theoretically provide the necessary regulation to control the inflammatory process.

In this study, we showed that MoDCs are an important source of IL-10 in cutaneous leishmaniasis. Previous studies have documented that the macrophages and regulatory T cells (CD4^+^ CD25^+^ Foxp3^+^) are the main source of IL-10 in the lesions and in supernatants of PBMC from patients with CL [43–45]. IL-10 may act in the control of cell-mediated lesion development in leishmaniasis [46, 47]. In mucosal leishmaniasis (ML) there is a lack of IL-10 response, in part explained by the downregulation of IL-10 receptor [48].

It has been demonstrated that PD-L1 and PD-L2 molecules induce CD4^+^CD25^+^ regulatory cells [49, 50]. PD-L1 and PD-L2 are able to control DC activation during antigen presentation through a variety of mechanisms [51, 52]. A study published by Wang et al. [53] showed that the upregulation of PD-L1 molecule was independent of IL-10 production, suggesting that there are other mechanisms of immune response to pathogens. In leishmaniasis, it has been demonstrated that PD-L1 and PD-L2 have distinct roles in regulating immunity to infection and that they are associated with the outcomes of infection. PD-L1 was associated with resistance and PD-L2 with susceptibility to mice infection with* L. mexicana *[54]. Studies about PD-L1 and PD-L2 are still controversial and have been performed basically using murine models.

The regulatory role of DCs in the overall immune response against parasitic worms is still unclear. It is known that worms promote local immunosuppression in the host, allowing the parasite to achieve long-term survival, which is usually associated with chronic infections [55]. Li and colleagues (2011) described a subset of DC that occurs naturally with regulatory activity in a murine model of* Heligmosomoides polygyrus* infection. These protective regulatory DCs promoted* in vitro* differentiation of Treg cells [56]. However, more studies are needed to understand the mechanisms that lead DCs to present regulatory functions.

Regarding the frequency of cells expressing the IL-10 receptor (IL-10R), in this study we showed a higher expression of this molecule in cultures stimulated with Sm29, even in the presence of SLA. Faria and colleagues (2005) demonstrated that the impaired expression of IL-10R in lesions from patients with ML was associated with the exacerbated immune response observed in this clinical form of disease. Other studies have associated a decrease in the expression of IL-10 receptor with parasite persistence and with an increase in the healing time of lesion [57–59].

## 5. Conclusion

Our study indicates that the* S. mansoni *antigen Sm29 has the potential to induce a desired regulatory response in CL patients. Recombinant Sm29 induced higher frequency of IL-10 and IL-10R on MoDCs compared to the unstimulated cell cultures. This molecule has a great potential to be used as a therapeutic agent to modulate inflammatory diseases. Our results may contribute to the development of new strategies for the treatment of diseases that are caused by excessive or inappropriate activation of the immune response, such as leishmaniasis.
